# The Role of Network Science in Glioblastoma

**DOI:** 10.3390/cancers13051045

**Published:** 2021-03-02

**Authors:** Marta B. Lopes, Eduarda P. Martins, Susana Vinga, Bruno M. Costa

**Affiliations:** 1 Center for Mathematics and Applications (CMA), FCT, UNL, 2829-516 Caparica, Portugal; 2 NOVA Laboratory for Computer Science and Informatics (NOVA LINCS), FCT, UNL, 2829-516 Caparica, Portugal; 3 Life and Health Sciences Research Institute (ICVS), School of Medicine, University of Minho, Campus de Gualtar, 4710-057 Braga, Portugal; id8266@alunos.uminho.pt (E.P.M.); bfmcosta@med.uminho.pt (B.M.C.); 4 ICVS/3B’s—PT Government Associate Laboratory, 4710-057/4805-017 Braga/Guimarães, Portugal; 5 INESC-ID, Instituto Superior Técnico, Universidade de Lisboa, 1000-029 Lisbon, Portugal; susanavinga@tecnico.ulisboa.pt; 6 IDMEC, Instituto Superior Técnico, Universidade de Lisboa, 1049-001 Lisbon, Portugal

**Keywords:** network analysis, differential network expression, model regularization, causal discovery, multi-omics, biomarker selection, precision medicine, personalized therapy

## Abstract

**Simple Summary:**

Knowledge extraction from cancer genomic studies is continuously challenged by the fast-growing technological advances generating high-dimensional data. Network science is a promising discipline to cope with the resulting complex and heterogeneous datasets, enabling the disclosure of the molecular networks involved in cancer development and progression. We present a narrative review of the network-based strategies that have been applied to glioblastoma (GBM), a complex and heterogeneous disease, along with a discussion on the relevant findings and open challenges and future research opportunities.

**Abstract:**

Network science has long been recognized as a well-established discipline across many biological domains. In the particular case of cancer genomics, network discovery is challenged by the multitude of available high-dimensional heterogeneous views of data. Glioblastoma (GBM) is an example of such a complex and heterogeneous disease that can be tackled by network science. Identifying the architecture of molecular GBM networks is essential to understanding the information flow and better informing drug development and pre-clinical studies. Here, we review network-based strategies that have been used in the study of GBM, along with the available software implementations for reproducibility and further testing on newly coming datasets. Promising results have been obtained from both bulk and single-cell GBM data, placing network discovery at the forefront of developing a molecularly-informed-based personalized medicine.

## 1. Molecular Networks in Precision Oncology

The discovery and molecular characterization of cancer subtypes harboring distinct molecular features and heterogeneous treatment responses comprise a crucial step in cancer treatment and management, as different cancer subtypes may respond differently to available treatment options [1]. Similarly, the stratification of tumor cells into distinct subpopulations provides valuable insights into the molecular drivers of intratumor heterogeneity, a major issue responsible for treatment failure and drug resistance and compromising personalized-medicine approaches [2,3]. Remarkable advances in the field have been reached thanks to the growing availability of molecular data delivered by next-generation sequencing (NGS) technologies, which combine, for a given patient, high-dimensional data from multiple omics platforms. In most statistical and machine learning-based tools that have been proposed to this end, molecular phenotypes are treated as single, non-interacting entities in the global molecular network context.

Network science has been widely used in the study of the topology and community structures of real-world networks [4]. In the biomedical domain, network science has emerged as a powerful tool to study biological systems governed by complex networks. Biological networks remain as one of the most studied classes of networks. Biological systems can be represented by networks, with nodes represented by distinct elements (e.g., genes, proteins, metabolites) and edges the interaction between the elements. Examples of biological networks are gene regulatory networks, gene coexpression networks, protein-protein interaction networks, signaling, and metabolic networks. Network analysis extends the identification of individual key elements in the biological network to disclosure sub-networks within the global complex network and how they relate to function and disease. Understanding how these complex networks function has important clinical implications, in particular in the definition of molecularly-informed diagnostic, prognostic, and disease prevention strategies.

The rise of network science in cancer genomics has opened new avenues for the identification of the networks involved in cancer processes, by the identification of the molecular network structures involved in cancer development and progression. Such network-based information can be further used to drive predictive models for future outcomes to more biologically meaningful solutions, towards a more personalized clinical and therapy decision, perfectly in line with the precision medicine framework.

**Figure 1:**
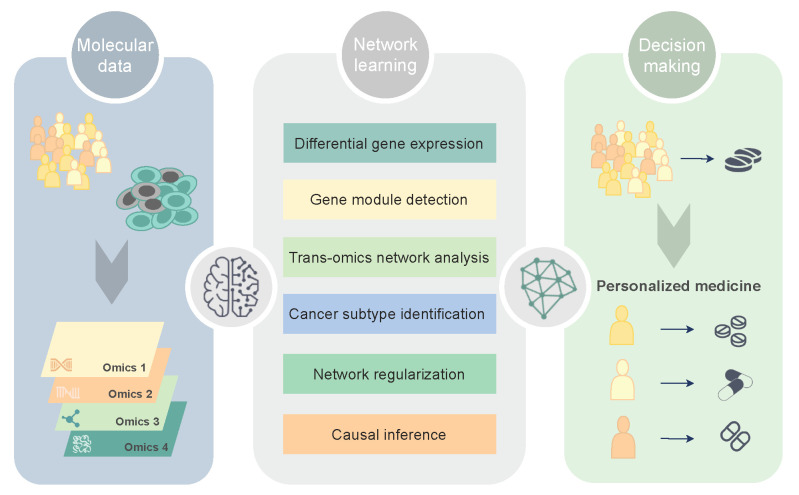
Overview of the current strategies in precision medicine for network learning in glioblastoma.

**Figure 2:**
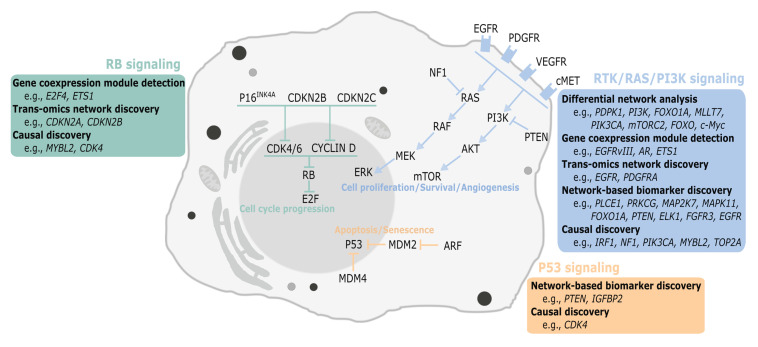
Simplified summary of major signaling pathways in glioblastoma and examples of genes previously identified by various network analyses.

In this narrative review, we cover many aspects of network science and examples of applications in the study of glioblastoma (GBM), a type of cancer characterized by a marked spatial and temporal heterogeneity at both the cellular and molecular levels [5]. Among the network-based strategies proposed to model GBM molecular data, particular attention is given to critical steps in GBM molecular characterization and stratification, namely differential network analysis, gene coexpression module detection, trans-omics network discovery, cancer subtype identification, biomarker discovery, and causal inference (Figure 1). A list of the computational tools and biological databases generated from the studies covered is also provided (Table 1), as well as a summary of the key findings and major signaling pathways involved in GBM (Figure 2). For the literature search, we used an automated search via the digital libraries of two major databases (Google Scholar and Web of Science), using several search string combinations of relevant keywords. A subsequent manual selection of the relevant studies for the scope of the review was performed.

## 2. Network Discovery in Glioblastoma

GBM is the most frequent primary malignant brain tumor in adults [1] and one of the most aggressive cancers [6,7], with a median survival of approximately 15 months after diagnosis [8,9]. GBM exhibits extensive tumor heterogeneity, both within and between tumors [6], which poses major challenges to diagnosis and treatment [10].

Verhaak et al. (2010) [1] performed multi-omics profiling of GBM tumors to disclose the molecular nature of GBM heterogeneity. The analysis revealed four major molecular subtypes based on transcriptomics, named classical, neural, proneural, and mesenchymal, which have recently been revised to three, classical, proneural, and mesenchymal [11]. The formerly identified neural subtype was not detected in the latter study, but it has rather been associated with tumor margin and normal neural lineage [12], probably explained by sample contamination with normal tissue in the first analysis [11]. Each identified molecular subtype is characterized by a panel of genetic alterations; in a simplistic manner, classical, mesenchymal, and proneural GBM subtypes are characterized by modifications in *EGFR*, *NF1*, and *PDGFRA/IDH1*, respectively [1]. This molecular classification of GBM impacts clinical outcome, as patients with primary and/or recurrent mesenchymal GBM commonly present a particularly dismal survival compared to non-mesenchymal cases [11]. Proneural GBM patients have a trend of increased survival, but do not benefit from intensive therapies, contrary to classical and mesenchymal GBMs [1]. Besides the associations between the molecular subtype and therapy response, *MGMT* methylation—a known predictive biomarker of response to standardized temozolomide treatment [13]—was not associated with the transcriptional subtypes [1]. Upon GBM recurrence, the majority of the cases maintained the same molecular subtype signature as the primary tumor, particularly in the case of mesenchymal GBM. This notwithstanding, a significant proportion of tumors switched molecular subtype after treatment, with an increment of proneural and mesenchymal GBMs [11]. Despite all the efforts to find similarities among the vast heterogeneity of GBMs, single-cell RNA sequencing analyses later performed by Patel et al. (2014) [10] revealed an even more complex level of heterogeneity in GBM, in which particular individual tumors contained distinct cells with transcriptional profiles resembling the various molecular subtypes firstly identified by Verhaak et al. (2010) [1]. Thus, while a predominant molecular subtype can be identified with bulk data generated at the population level, more refined single-cell sequencing analyses identify multiple clones associated with different GBM molecular subtypes in a single tumor. In addition, this study also found the existence of “hybrid” cells, i.e., individual cells with the representation of more than one subtype [11]. Interestingly and even though patients whose GBM is classified as proneural present a trend of increased survival [1], when this subtype is subdivided into pure proneural, low-heterogeneity proneural, and high-heterogeneity proneural, the increased heterogeneity of this class lowers the survival of patients, highlighting the clinical relevance of intratumoral heterogeneity [10]. Overall, these findings clearly highlight the remarkable level of complexity regarding the heterogeneity of GBM, potentially applicable to other cancer types. Network discovery is particularly relevant in the case of highly heterogeneous cancers, for which network-based machine learning from large-scale and multi-level NGS data at both bulk and single-cell levels is expected to provide a deeper insight into GBM’s molecular mechanisms of disease development and progression.

### 2.1. Differential Network Analysis

One of the most common tasks in genomics is the identification of differentially expressed genes (DEG) across different disease conditions or cell types. This practice, although of recognized value for biomarker discovery and the development of targeted therapies, misses the overall network information genes carry while co-expressed in common regulatory pathways. To fill this gap, the differential network analysis in genomics (DINGO) model [14] (Table 1) was proposed for estimating group-specific networks and inferring differential networks. The method jointly estimates the group-specific conditional dependencies by decomposing them into global and group-specific components.

In the context of GBM, DINGO was applied to TCGA data from multiple omics platforms (mRNA expression, DNA copy number, methylation, and microRNA expression) with the goal of building differential networks for long- and short-term survivors (LTSs and STSs, respectively). The application focused on selected key signaling pathways (RTK/PI3K, p53, and Rb), involved in cell migration, survival, and apoptosis and closely associated with GBM biology.

The differential networks between GBM LTSs and STSs estimated from the different omics platforms revealed many hub genes with several important roles in GBM progression, e.g., via a connection with the *c-Myc* gene, associated with GBM proliferation and decreased survival. Amongst the differentially expressed hub genes, *PDPK1*, detected in the DNA copy number network, promotes *EGFR* activation [15], a known driver of GBM cell proliferation, invasiveness, motility, angiogenesis, and apoptosis evasion [16], contributing to GBM’s aggressiveness. *PI3K* genes identified from mRNA expression, DNA copy number, and methylation are frequently altered in GBM (88% of GBMs present alterations to the RTK/RAS/PI3K axis) [17], supporting pro-tumor effects such as proliferation, growth, differentiation, survival, and angiogenesis [18,19]. From the DNA methylation data, the hub *FOXO1A* gene was associated with *MLLT7* (*FOXO*) and with *PIK3CA* (*PI3K*) [14]. The regulatory networks including *mTORC2*, *FOXO*, and *c-Myc* are highly intercorrelated with a shorter survival of GBM patients [20]. A previous study had already demonstrated the following connections: by acetylation, mTORC2 is able to impede FoxO activity that could surpass PI3K/AKT inhibition, upregulating c-Myc. In the clinical setting, acetylated FoxO and c-Myc were highly correlated in GBM samples, and patients expressing high levels of c-Myc showed a significantly shorter overall survival [20]. Reports on in vivo data additionally suggest c-Myc as a potential therapeutic target in GBM [21] and as an important player in PARP inhibition resistance [22]. The hubs from microRNA expression revealing a higher degree of connectivity were hsa-miR-103 and hsa-miR-107 [14], which directly interact with the *CDK5R1* 3’UTR [23], an activator of *CDK5* [24], which is overexpressed and oncogenic in GBM [25,26,27,28,29,30].

### 2.2. Gene Coexpression Module Detection

Uncovering gene coexpression modules, or groups of highly coexpressed genes, has been pointed out as a promising therapy research direction for the identification and validation of novel molecular targets, through understanding of how gene modules interact with cancer lesions.

Horvath et al. (2006) [50] performed gene expression profiling on RNA from 120 GBM patient samples from two independent datasets. The identification of gene modules was performed via weighted gene coexpression network analysis (WGCNA) [37,51] (Table 1), by constructing a weighted gene coexpression network based on the pairwise Pearson correlations between the gene expression profiles, followed by an unsupervised hierarchical clustering to detect groups of highly coexpressed genes. For the first GBM dataset, the authors detected five gene coexpression modules, which were significantly enriched for genes across the ontological classes mitosis cell cycle, immune response, neurogenesis, cytoplasm, and metabolism. The same five gene modules were obtained for the second dataset, after weighted gene coexpression network construction based on the genes found for the first dataset, indicating that gene coexpression modules are highly preserved in both GBM datasets.

Among the various findings of the study, the analysis identified a mitosis/cell cycle module in GBM that is downstream of the mutant epidermal growth factor receptor, *EGFRvIII*, as shown by studies done in an isogenic model system. Furthermore, *ASPM* (abnormal spindle-like microcephaly associated) was identified as a key gene in the mitosis/cell cycle module, being associated with increased cell proliferation, underexpressed in normal tissue compared to GBM. The results also suggested *ASPM*’s involvement in GBM pathogenesis by promoting a stem cell phenotype, therefore supporting *ASPM* as a potential GBM molecular target [50]. Shortly after, *ASPM* was associated with glioma grade, showing increased expression from non-tumoral samples to grade II, III, and IV gliomas [52,53]. More recently, *ASPM* was also associated with the survival of patients with GBM [50,54] and glioma [55], indicating that the hub genes identified by Horvath et al. (2006) [50] might be new prognostic biomarkers. Interestingly, *ASPM* was previously identified to be expressed at higher levels in secondary GBMs as compared to lower grade astrocytomas [56] and was further validated in another study as one of the six genes with the highest relative expression in GBM compared to pilocytic astrocytomas [57]. *ASPM* also emerged as a hub gene in additional screening studies in GBM [55,58,59], and its functional relevance was recently validated in vitro and in vivo [55]. Specifically, *ASPM*-silenced GBM cells presented decreased proliferation rates and formed in vivo subcutaneous tumors with significantly reduced sizes. Mechanistic assays suggested an association between ASPM and the Wnt/β-catenin pathway, highly relevant in GBM, and that *ASPM* knockdown was linked to a G0/G1 arrest of GBM cells [55]. Globally, this is a paradigmatic example on the use of network analysis that allowed the identification of a novel GBM biomarker, with critical oncogenic functions and prognostic clinical potential, for which novel smart therapies can be developed in the future.

Since it was first proposed, WGCNA has been extensively applied in subsequent studies on the gene coexpression relationships across glioma subtypes to identify key genes and pathways associated with the occurrence, progression, and survival of GBM (e.g., [60,61,62,63,64,65,66,67,68]).

Disease module extraction (DiME) is another algorithm to identify disease modules from biological networks [38] (Table 1), built up the community extraction (CE) module criterion originally proposed for social network analysis [69]. In a study of the molecular mechanisms involved in glioma progression, Liu et al. (2014) [38] developed novel heuristics to optimize CE and incorporated the B-score, a measure of statistical significance, to evaluate the quality of the modules extracted from low- (grade II) and high-grade (GBM) glioma co-expression networks.

The datasets used consisted of microarray expression data from 97 grade II patients and 126 GBM samples, retrieved from the Rembrandt database (Table 1). Further validation was performed on the TCGA GBM dataset and the grade II glioma expression dataset (GSE30339) from the Gene Expression Omnibus (GEO) database (Table 1), consisting of 197 and 23 samples, respectively. From grade II gliomas to GBM, a shift in network topology was observed, with GBM tumors showing altered levels of transcripts involved in extracellular matrix (ECM) reorganization and angiogenesis, which stand as markers of a more aggressive phenotype. The analysis also identified several statistically significant modules that were reproducible across the different datasets, from which the transcription factors *E2F4*, *AR*, and *ETS1* are highlighted as potential key regulators in tumor progression. *E2F4* is overexpressed in high-grade gliomas (HGGs) and is associated with poorer survival outcome [70]. Interestingly, AR was later found overexpressed in GBMs [71,72,73] and associated with poor survival of patients with low-grade gliomas (LGGs) [72]. A growing body of evidence also suggests AR as a modulator of treatment response in GBM, for example: (i) AR confers radiation resistance [73]; (ii) the nuclear translocation of AR is affected by cedrol treatment, suppressing GBM progression [74]; (iii) the 5α-reductase enzyme inhibitor dutasteride in combination with AR antagonists effectively decreased the proliferation of GBM cells [75]; and (iv) a curcumin analog induced the ubiquitination of AR and consequent degradation, suppressing the growth of temozolomide-resistant cells [76]. Similarly, ETS1 had also been studied for its impact in the expression/regulation of other genes in brain tumors, such as *uPA* and Flt-1/VEGFR-1 in astrocytic tumors [77,78] and *DPP-III* when conjugated with Elk-1 in human GBM [79]. More recently, ETS1 was also found to transcriptionally activate the C250T mutant form of the *TERT* promoter [80], which has been recognized as a critical driver of replicative immortality in GBM [81,82].

To identify gene expression relationships across multiple human tumors, Dunwoodie et al. (2018) [83] used the Knowledge-Independent Network Construction (KINC) software [39] (Table 1) to build two RNAseq-based gene coexpression networks based on publicly available datasets from TCGA (GBM; LGG; bladder urothelial carcinoma; thyroid carcinoma; and ovarian serous cystadenocarcinoma) and the NCBI Sequence Read Archive (SRA) (GBM, normal brain, and Parkinson’s brain) (Table 1). KINC uses Gaussian mixture models (GMMs) before applying pairwise correlation analyses to the gene expression matrices. The major feature of KINC is its ability to deconvolute mixed-condition expression patterns (e.g., mixed tumor expression profiles from GBM and non-GBM samples) without the need to separate gene expression profiles before analysis.

A 22-gene GBM-specific module was identified in the two gene expression sources. The module showed increased RNA expression compared to normal brain and LGG samples and decreased DNA methylation in GBM in relation to LGG. Moreover, seventeen of the 22 shared genes were found in the Glioblastoma Bio Discovery Portal (GBM-BioDP) (Table 1) based on results from Verhaak et al. (2010) [1]. The highest expression for these genes was observed for the mesenchymal subtype, and high expression was associated with decreased survival in each GBM subtype. Four genes (*SIGLEC9*, *MYO1F*, *LAPTM5*, *ITGB2*) from the 22 gene list were upregulated in mesenchymal GBM. Interestingly, recent data indicated *ITGB2* as being highly positively correlated with the tumor-associated macrophage (TAM) marker *CD68* [84], suggesting an increased infiltration of TAMs in *ITGB2*-positive gliomas. Furthermore, higher expression of *ITGB2* was associated with shorter overall survival of glioma patients [84]. These data are in agreement with the study of Wang et al. (2017) [11], in which the authors identified an increment of infiltrating macrophages in mesenchymal GBMs and shorter survival of this subtype [11].

### 2.3. Trans-Omics Network Discovery

The rapid advances in sequencing technologies have enabled the acquisition of patient molecular information by diverse omics assays. With the great amounts of molecular information generated per patient, it became possible to explore the relationships among molecular features across different omics layers, e.g., DNA variants and downstream phenotypes, and potentially identify trans-omics therapeutic targets.

The first method we showcase extends DINGO (Section 2.1) to integrate matrices of expression data on the same samples from different platforms (iDINGO) [85] for a deeper biological understanding of the relationships across the omics layers. The method estimates group-specific dependencies and makes inferences on the integrative differential networks, considering the biological hierarchy among the platforms.

Jörnsten and co-authors (2011) [86] addressed the challenge of uncovering how DNA copy number aberrations (CNAs) affect gene expression, via endogenous perturbation analysis of cancer (EPoC), a method that constructs mRNA-based network models in which mRNA patient profiles are regarded as responses to perturbations induced by CNAs during tumor evolution. The goal is to detect disease-driving CNAs, assess their effect on target mRNA expression, and disclose GBM biomarkers, which are predictors of patient survival.

To identify the transcriptional and CNA–mRNA couplings, EPoC solves the two complementing linear systems, one representing the transcriptional interaction network and the other the CNA-driven network, consisting of CNA–mRNA interactions. EPoC first uses a combination of lasso regression and bootstrapping to estimate the parameters of the EPoC network model from paired DNA- and RNA-level data. Then, a score based on sparse singular-value decomposition of the derived CNA–mRNA network model is computed to disclose prognostic biomarkers able to stratify patients into LTSs and STSs.

The method was applied to TCGA GBM datasets (CNA and mRNA), based on 10,672 genes and 186 patients. Known disease-driving CNAs controlling multiple downstream hub genes could be identified, namely oncogenes and tumor suppressors with a known role in GBM, including *EGFR*, *PDGFRA*, *CDKN2A*, and *CDKN2B*, but also other interesting hub genes not previously associated with GBM, e.g., *MTAP* and *SEC61G*. The model also detected robust CNA–mRNA links between the hubs *EGFR*, *PDGFRA*, and *CHIC2* and markers of early neural development, such as a GBM stem cell marker, *CD133* (*PROM1*), and the transcription factors *SOX10*, *SOX11*, *NR2E1* (*TLX*), and *NKX2.2*. From all these pinpointed genes, the impact of *MTAP*, *SEC61G*, *CD133*, and *SOX11* was previously evaluated in GBM patients. *MTAP* has been implicated as a prognostic factor, despite discordant findings: *MTAP* deletion was associated with decreased progression-free survival in GBM patients [87], while another study reported that adult GBM patients lacking *MTAP* expression had better survival than those with detectable levels of *MTAP* expression [88]; *SEC61G*’s higher expression was linked to decreased overall survival of patients from TCGA [89]; SOX11 overexpression was reported to be associated with longer overall survival [90,91]; finally, the impact of CD133 in patient prognosis is quite controversial, but a recent meta-analysis of published data concluded that higher expression of CD133 is associated with poorer prognosis [92]. The results obtained by the CNA-driven GBM network model were further validated in four GBM cell lines (T98G, U-87MG, U-343MG, and U-373MG), via perturbations on *NDN*, which encodes the p53-interacting protein necdin, to suppress GBM cell growth. In the resulting CNA-driven network, *NDN* shares a target, fibroblast growth factor 9 (*FGF9*), with *PDGFRA*, which is frequently amplified in GBM.

Multiple microRNAs (miRs) have been described as promoting or suppressing oncogenesis, as modulators of gene expression. Genovese et al. (2012) [93] inferred putative GBM regulatory networks between miRs and mRNA GBM data using the context-likelihood-relatedness (CLR) [46] algorithm. The dataset counted for 290 matched miR and mRNA expression profiles, yielding a total of 26,297 edges between 254 miR and 6152 mRNA.

CLR generates pair-wise associations based on mutual information (MI). The algorithm involves a statistical background correction step, in which the significance of each miR-mRNA MI value is assessed against the background distribution of MI scores for all possible miR/gene pairs that include either the miR or its target. After this background correction, the most probable interactions are those whose MI scores stand signficantly above the background distribution of MI scores. The analysis suggested a role of mRNA regulation by miR in the definition of the molecular proneural and mesenchymal subtypes, revealed by the marked expression differences in the edges obtained for the global network between these subtypes. *miR-34a* expression was found to be prognostic in GBM, in both TCGA and an independent cohort of human gliomas, with *miR-34a* low-expressing GBM patients exhibiting an overall improved survival. The same results were obtained for the proneural subtype. Evidence of *miR-34a* as a tumor suppressor in proneural GBM was also obtained by integrative in silico analyses, functional genetic screening, and experimental validation [93]. A direct regulation of *PDGFRA* by *miR-34a*, previously found as functionally related to proneural GBM [94], was also confirmed. Additionally, promoter enrichment analysis of the edges identified uncovered a novel regulation of TGFβ signaling via a Smad4 transcriptomic network by *miR-34a* [93], later shown to be implicated in the regulation of GBM cells’ migration and invasion [95].

### 2.4. Network-Based Learning

#### 2.4.1. Cancer Subtype Identification

Cancer genomic profiling has been extensively used to stratify cancer samples into well-characterized molecular subtypes towards improved diagnosis and prognosis. The increasing availability of information from biological networks from multiple molecular assays has motivated researchers to incorporate such invaluable information into the learning procedure.

Xu et al. (2016)  [40] addressed the problem of identifying cancer subtypes, by developing the weighted similarity network fusion (WSNF) method (Table 1). WSNF is based on a previously proposed method, similarity network fusion (SNF) [96], a multi-omics data fusion method that stratifies patients into cancer subtypes based on a fusion similarity matrix obtained from the similarities between patients from different data types and a subsequent clustering step. WSNF extends SNF to make use of available information on biological networks, such as gene regulatory networks, by incorporating feature weights (i.e., feature importance) into the clustering process for cancer subtype identification from multi-omics data, which are obtained by combining features’ ranking in the network and their expression variation.

In an application to the TCGA GBM dataset, WSNF was built on gene expression data and information from a complex miRNA-TF-mRNA regulatory network, with the nodes representing the features, i.e., miRNA, transcription factors (TF), and mRNAs, and the edges of the interactions between them, the latter retrieved from interatomic databases. In this study, WSNF was able to identify three GBM subtypes with significantly different survival patterns and enriched pathways.

GBM subtype identification was also approached via CSPRV (cancer subtype prediction using $RV_{2}$)  [41], a method that overcomes previous methods also integrating multi-source transcriptome expression and biological networks for cancer subtype prediction, by considering the data-view weights in data integration, the importance of features, and their relationships in data integration. CSPRV is a multi-step procedure that first extracts multiple expression features of each regulatory element based on the regulatory associations in the biological networks; then, it reduces the high-dimensional expression features extracted to a low-dimensional space and constructs an integrative feature matrix for each sample; it uses a matrix correlation method, $RV_{2}$ [97], to predict the similarities between samples in each expression data-view, which are then fused according to the different integration weights; and finally, it clusters samples into different groups based on the integrated similarities between samples. CSPRV showed improved performance in the identification of GBM subtypes with different survival patterns over WSNF.

#### 2.4.2. Model-Based Biomarker Discovery

One of the biggest challenges in the identification of predictive models for cancer outcomes and key drivers of disease processes is the problem of the high-dimensionality of molecular data matrices. The incorporation of model regularizers in generalized linear regression frameworks has shown promise in both predictive model performance and feature selection across many biomedical contexts (see [98]). These include the least absolute shrinkage and selection operator (lasso) [99], elastic net [100], fused lasso [101], along with variants such as adaptive lasso [102] and group lasso [103]. However, these strategies rely solely on data and the computational aspects of algorithm implementation, without incorporating any prior information on the biological processes.

The remarkable technological advances in the last few decades and great efforts of large consortia, e.g., through genome- and proteome-wide projects, have produced a vast amount of molecular and pathway information now available through biological databases (Table 1). Regulatory networks can be represented by graphs, where the nodes or vertices represent the genes or gene products and the edges the relationships among them. To take advantage of such a massive amount of valuable information, several methods have been proposed to incorporate information from these graphs, e.g., pathway topology, into model generation, leading to more interpretable solutions.

Li and Li (2008) [104] introduced a network-constrained penalty function to identify genes and subnetworks that are related to phenotype responses. The regularizer penalizes the $\ell_{1}$-norm (lasso) of the coefficients while encouraging the smoothness of the coefficients on the network through a graph Laplacian constraint. The proposed network-constrained procedure was applied to a GBM microarray gene-expression dataset [104] on 50 patients and 1533 genes found in the Kyoto Encyclopedia of Genes and Genomes (KEGG) network of 33 pathways. The method was successful in the identification of subnetworks of the KEGG pathways that were related to patient survival from GBM, some of them supported by previous studies. Among the subnetworks identified, the largest included genes involving the MAPK signaling pathway (e.g., *PLCE1*, *PRKCG*, *MAP2K7*, *ZAK*, *KBKG*, *TRAF2*, and *MAPK11*) and its connected pathways, such as the PI3K/Akt signaling pathway (e.g., gene *GYS1*) and its target *FOXO1A* [105,106]. Another subnetwork identified four genes, *PTEN*, *PRKG2*, *MAPK8IP2*, and *ELK1*. PTEN is a negative regulator of PI3K signaling [107,108], frequently dysregulated in GBM; the *PRKG2* gene encodes cyclic guanosine monophosphate (cGMP)-dependent protein kinase II and is responsible for anti-proliferative signals in human gliomas [109]; the *MAPK8IP2* gene encodes a JNK-interacting protein, influencing JNK and p38MAPK signaling [110], which are pivotal in GBM [111,112]; and ELK1 is a downstream target of PKCη-, and its activation results in increased proliferation in GBM cell lines [113]. The gene pairs subnetworks also revealed genes with a previously reported role on GBM, namely the *CTNNB1* [114,115], *CTLA-4* [116], and *CLDN* gene families [117,118].

Other strategies for accounting for network-based information during the learning process encompass the introduction of weights in the coefficients, which can be derived either from the network topology of external databases (e.g., protein-protein interaction databases) or of the data network structure itself, e.g., the correlation/covariance matrices, as shown in survival and classification on breast, melanoma, and ovarian cancer datasets [42,43,119,120,121], using available implementations in the R packages glmnet [122] and glmSparseNet [42] (Table 1). Considering the elastic net regularizer, this is equivalent to adding a weight factor to the penalty term.

Under the regularized-based learning framework, a variant of the above correlation-based regularizers, the twin networks recovery (twiner) penalty was introduced by Lopes et al. (2020) [123,124] to explore the differences in the gene correlation networks in a classification setting. Given two disease types, the goal is to select features that have a similar correlation pattern across the two conditions, which can be regarded as putative disease targets for the development of shared therapeutic approaches for the two diseases. The twiner regularization weights are built based on the similarities given by pairwise correlations between the variables independently obtained from two given datasets (disease types/conditions). For a given variable (gene), the lower the distance between the correlation vectors in the two disease conditions, the lower the penalty induced on that gene. These ideas have been recently extended to survival modeling via the TCox [125], intended to select variables with distinct correlation patterns across two group conditions, e.g., cancer vs. normal tissue, which have a relation to the survival outcome.

The suitability of the above network-based regularizers has been shown to be promising when tackling GBM heterogeneity via biomarker selection and classification [123] in a single-cell RNA sequencing (scRNA-seq) GBM dataset [126]. The authors proposed a classification setting through sparse logistic regression to classify cells into different populations (neoplastic and normal cells), while selecting gene features discriminating between classes, but also those shared by different neoplastic clones (tumor core and infiltrating cells), standing as putative therapeutic markers to target multiple clones.

Among the genes revealed by the analysis, several have a known role in GBM, in particular: three upregulated genes in GBM infiltrating tumor cells with known functions involving the invasion of the interstitial matrix, *ATP1A2*, *PRODH*, and *FGFR3* [126]; the epidermal growth factor receptor (*EGFR*), upregulated in neoplastic periphery astrocytes and significantly mutated in GBM [127,128]; *ANXA1*, upregulated in infiltrating astrocytes, promoting GBM tumor growth and progression [129] and correlated with *IGFBP2*, also selected and upregulated in GBM; the serine protease *HTRA1*, downregulated in neoplastic periphery astrocytes, a binding partner of the macrophage migration inhibitory factor (*MIF*) [130]; *SOX9* and *EGFR*, associated with astrocyte development and differentiation; *CHCHD2*, showing coamplification with the well-known GBM marker, *EGFR*, in glioma [131,132]; the transcriptomic factor *SOX9*, involved in brain development and lineage specification, with an established oncogenic role in gliomas [126,133]; *PSAP*, a target for glioma treatment, by promoting glioma cell proliferation via the TLR4/NF-kB signaling pathway [134]; *PREX1* and *ABHD2*, shown to promote tumor invasion and progression in GBM [135]; and the tumor suppressor *BIN1*, regulated by *HNRNPA2B1* and a putative proto-oncogene in GBM [136]. The relevance of the genes identified was confirmed by their significance in the survival outcomes from a bulk GBM RNA-seq dataset (TCGA), as well by their association with several Gene Ontology (GO) biological process terms.

### 2.5. Causal Discovery

The discovery of the causal relationships in complex molecular networks is a challenging problem that has recently attracted many researchers in the field of cancer genomics, to overcome the fact that measures of association do not reveal the direction of the estimated association. The identification of causal relationships within molecular paths is crucial to unveil cancer disease dynamics and therapeutic targets.

Zhang and co-authors (2018) [45] extended module detection and trans-omics network discovery (see Section 2.2 and Section 2.3) to disclose the causal role of long non-coding RNAs (lncRNAs) on messenger RNAs (mRNAs). lncRNAs interact with mRNAs through gene regulatory networks to carry out biological functions, such as cell differentiation, cell proliferation, and cytoprotective programs [137]. The authors proposed module-specific lncRNA-mRNA causal regulatory networks (MSLCRN) (Table 1), a method aimed at inferring and analyzing module-specific lncRNA-mRNA causal regulatory networks.

MSLCRN is a three-step procedure to infer module-specific lncRNA-mRNA causal regulatory networks. The method first identifies lncRNA-mRNA coexpression modules using WGCNA [37]. Second, the causal effect of lncRNA on mRNA in the causal pairs in each module identified is estimated using the intervention calculus when the DAG is absent (IDA) algorithm [138], in two steps: (i) using the parallel-PCalgorithm [44,139] (Table 1), a parallel version of the PC algorithm [140], to learn the causal structures, or directed acyclic graphs (DAGs), where the nodes are the lncRNAs or mRNAs and the edges between two nodes represent the causal relationship between them; and (ii) estimating the causal effects of lncRNAs on mRNAs by applying do-calculus [141]. A global lncRNA–mRNA causal regulatory network was finally identified, by integrating the module-specific lncRNA–mRNA causal regulatory networks.

In an application of MSLCRN to matched expression data of 9704 lncRNAs and 18,282 mRNAs in 451 GBM samples [142], twenty-three lncRNA-mRNA causal regulatory networks were identified. In subsequent Gene Ontology (GO) and KEGG enrichment analyses to assess the biological meaningfulness of the MSLCRN networks identified, fifteen out of the 23 (65.2%) networks were found significantly enriched in at least one GO biological process or KEGG pathway.

Plaisier et al. (2016) [143] developed the TF-target gene interaction database and the Systems Genetics Network Analysis (SYGNAL) pipeline (Table 1), for dissecting GBM-related causal transcriptional regulatory networks (TRNs) and predicting drug targets, based on multi-omics and clinical patient data. The SYGNAL pipeline uses correlative, causal, and mechanistic inference approaches to infer the causal flow from mutations to regulators (TFs and miRNAs) and their downstream target genes.

The authors hypothesized that a TRN describing TF-miRNA regulatory interactions would be helpful for prioritizing combinatorial interventions and drive therapeutic research to the development of therapies targeting TFs and miRNAs. Such a TRN is potentially more effective than single TF and miRNA targeted therapies, by the regulation of several genes involved in diverse oncogenic processes. The SYGNAL pipeline was applied to the multi-omics TCGA GBM data. The resulting gbmSYGNAL network predicted 67 novel regulators (TFs and miRNAs) associated with patient survival or oncogenic processes. Among the relevant findings of the gbmSYGNAL network, the network revealed modulation of *IRF1* by mutated *NF1* and *PIK3CA* to increase tumor lymphocyte infiltration.

More recently, Liu and co-workers (2020) [47] developed a computational pipeline, Integrative Modeling of Transcription Regulatory Interactions for Systematic Inference of Susceptibility in Cancer (inTRINSiC) (Table 1), to dissect the TF regulatory circuitry underlying the heterogeneity of GBM based on the integration of epigenomic, transcriptomic, protein interaction, and genetic perturbation. The inTRINSiC pipeline runs in two main steps. The first step encompasses procedures for constructing context-specific regulatory networks that readily accommodate additional mechanisms of transcription regulation. In the second step, context-specific networks are used to perform in silico perturbation analysis and infer the dependency on TFs by each subtype, through simulation of the information flow from the TF regulation layer to the protein signaling layer.

The platform was applied to sample data from the Cancer Cell Line Encyclopedia (CCLE) [35], TCGA, and the Chinese Glioma Genome Atlas (CGGA) project (Table 1), enabling the identification of the differential regulatory activity of TFs across different GBM subtypes. In the subsequent in silico perturbation analysis, the authors identified *MYBL2*, a member of the *MYB* family of transcription factors associated with cell cycle progression and maintenance of cells in an undifferentiated state [144], as a transcription factor essential for the proneural subtype, affecting patient survival, in which patients with low *MYBL2* expression showed significantly longer survival [47].

Bayesian networks (BNs) are a type of directed acyclic graph (DAG), where each node is a random variable and each edge represents a conditional probabilities relationship between a given pair of nodes. BNs can be used to infer regulatory networks, through the identification of the conditional probabilities, which in turn can be used to infer the direction of information flow within the network and hypothesize putative underlying causal relationships between the nodes. Kaiser et al. (2017) [145] used Bayesian inference to disclose changes in the extracellular regulatory network related to host immunity and co-occurring with oncogenesis, such as an increase in cell proliferation and epithelial-to-mesenchymal transition (EMT). The authors used TCGA gene expression data from tumor and normal breast cancer tissue samples, combined with defined gene signatures, the metagenes, which represented groups of genes, instead of single genes, associated with the processes of interest, either immune or cancer related. Although the study was developed for breast cancer data, the authors investigated other cancer types, namely GBM, to determine whether the relationships inferred were breast cancer-specific or not.

The Bayesian networks were generated from the metagene constructs using an incremental associated Markov blanket (IAMB) algorithm [146] implemented in the bnlearnR package (Table 1). The IAMB algorithm starts with a forward phase that generates a network by maximizing the conditional independence of the nodes, followed by a backward phase that removes one-by-one any remaining conditionally independent connections. The use of metagene constructs was intended to simplify the DAG, by reducing the dimension to a low number of less computationally expensive and interpretable nodes, and help eliminating noise in the data. Confidence for the edges was calculated using a bootstrap resampling strategy, by randomly sampling patient data with replacement, with a network generated from the new dataset in each replicate.

In the resulting breast and GBM networks, increased proliferation led to increases in M1 polarized macrophages associated with increases in natural killer cell infiltration in GBM. When using only cancer samples, however, the relationship between EMT and macrophage polarization in the network reemerged. The authors reasoned that these differences might be related to the fact that GBM arises in an immune-privileged area.

Kunkle et al. (2013) [147] developed a meta-analysis of 12 microarray studies on normal and astrocytoma tissues, followed by a Bayesian analysis of the results of this meta-analysis. The goal was to identify key genes and/or pathways in the development of astrocytic tumors. The analysis pipeline involved the identification of a significant set of de-regulated genes across the microarray studies considered, enrichment and network analyses of the significant genes and investigation and validation of the network analysis.

The Bayesian networks were inferred using the Banjo (Duke University, NC) software (Table 1), which performs Bayesian structure inference based on Dirichlet scoring. The Bayesian network genes in each stage of astrocytoma (grades I–IV) were used to identify key genes in the development of astrocytoma, through the identification of a set of Markov blanket genes from each gene network, which stands, for a given node, as the set of the parents, children, and the children’s parents. In the resulting GBM (grade IV) network inferred, the most influential genes, *COL4A1*, *EGFR*, *BTF3*, *MPP2*, *RAB31*, *CDK4*, *CD99*, *ANXA2*, *TOP2A*, and *SERBP1*, were able to accurately predict non-tumor and tumor cases [147]. Some of those genes have been reported to be highly expressed or activated in GBM, e.g., *EGFR* [17], BTF3 [148], *CD99* [149,150], TOP2A [151], and *SERBP1* [152]. Moreover, the analysis of gene-gene interactions revealed joint effects of changes in the expression of Markov blanket genes in the risk for developing GBM, which was dramatically increased with joint effects of four to 10 Markov blanket genes, resulting in a 9 to 85.9% increase, respectively, compared to the normal population [147].

In the BN research vein as well, Cai et al. (2019) [153] proposed a tumor-specific computational framework based on Bayesian causal modeling, tumor-specific causal inference (TCI), to estimate the causal relationships between somatic genome alterations (SGAs) and molecular phenotypes (e.g., transcriptomic, proteomic) observed in an individual tumor. TCI integrates multiple types of SGAs and molecular phenotypes to estimate which genome perturbations are causally influencing one or more molecular/cellular phenotypes in an individual tumor. TCI was applied to 5097 tumors across 16 cancer types in TCGA, including GBM, to derive tumor-specific causal network models. TCI identified 634 causative SGAs of cancer-related DEGs in a significant number of tumors. TCI inferred statistically robust causal relationships, which were further supported by computational and experimental analyses.

In another branch of causal discovery, Mendelian randomization (MR) uses genetic variants, such as single-nucleotide polymorphisms (SNPs), that are robustly associated with an exposure as proxies for the risk factor of interest [154], therefore eliminating confounding and reverse causation effects between the exposure of interest and outcome. Howell et al. (2020) [155] used MR to assess the causal relationship of associations of 36 reported glioma risk factors obtained from a systematic MEDLINE search on observational epidemiology studies. A meta-analysis of two glioma genome-wide association studies (GWASs) was then performed, and a two-sample MR analysis using the GWAS exposure and outcome datasets investigated the causal relationships between the risk factors identified and glioma incidence. The MR analysis revealed that four genetically predicted traits increased the risk of glioma, GBM, or non-GBM, namely longer leukocyte telomere length, liability to allergic disease, increased alcohol consumption, and liability to childhood extreme obesity. On the other hand, two traits, increased low-density lipoprotein cholesterol (LDLc) and triglyceride levels, decreased the risk of non-GBM cancers.

## 3. Major Challenges and Future Strategies

The enormous quantity of multi-omics data generated in recent cancer-related studies and the software tools able to perform network analyses portrayed in this review must be further explored, integrated, and made available to wider audiences. It is still crucial to better identify which genes involved in a particular network are truly clinically relevant. As denoted by the authors that developed the WGCNA software, the large list of hub genes identified in the analysis might be further shortened based on some filtering strategies, such as the availability of protein biomarkers and mouse models for validation, biological relevance based on ontology data, and the capacity to be used as a therapeutic target [50]. The information gathered from network analyses is expected to help in the prediction of tumor characteristics and, ultimately, assist in precision medicine approaches that might influence clinical decisions.

Having in mind the tremendous effect that these data can have in the clinical setting, the need for validation of the utility of particular networks is undeniable. Studies from Horvath et al. (2006) [50] perfectly described the workflow of the identification of a network to the clinical validation, from bioinformatics analyses to in vitro studies and the search for clinical significance. This study led to the identification of the *ASPM* gene [50], and its relevance was further validated both by the same authors and in other groups [50,55], as detailed in Section 2.2. Similarly, the functional relevance of *SERBP1*, identified in a Bayesian network [147], was further evaluated by an independent study [152].

The validation of molecular networks might be performed by testing key genes in genetically-manipulated GBM models, combined with pharmacological approaches for the activation/inhibition of the target, using both cell lines and patient-derived cultures, to evaluate functional effects and omics alterations. Complementing in vitro findings with in vivo models of GBM, including syngeneic and xenograft mice models, is also of critical translational relevance. In the last few decades, some novel in vitro and in vivo models have emerged, which attempt to surpass some of the limitations and simplicities of homotypic in vitro cultures with GBM cell lines. For example, organoids with heterotypic cultures can be grown from a patient tumor tissue and be used for therapy response evaluations [156]. Different studies showed that GBM organoids better recapitulate the parental tumor, maintaining the critical cellular and molecular heterogeneity and mutational profiles of particular tumor niches [157,158]. Beyond these advantages, organoids also allow the establishment of heterotypic cultures, in which non-tumor cells (i.e., immune cells, endothelial cells, etc.) that are commonly present in the original tumor can be co-cultured with exogenous cells, better mimicking the tumor microenvironment [159]. At the in vivo level, patient-derived xenografts (PDXs), in which a patient tumor is directly implanted in immunodeficient mice, have also emerged as a promising approach to better reflect the various aspects of tumor pathophysiology, heterogeneity, and response to therapy [160,161,162]. Orthotopic PDXs of GBM have been shown to preserve tumor morphology, invasion patterns, and critical molecular aberrations, such as alterations of *TERT*, *EGFR*, *PTEN*, *TP53*, *BRAF*, and *IDH1*. On the contrary, necrosis and microvascular proliferation seem to be lost upon engraftment, as is the case for some molecular alterations, including *PDGFRA* amplification [163]. PDXs represent excellent models to study and/or validate molecular networks, as they better maintain GBM heterogeneity and predict therapy responses. In fact, recent studies have been trying to find correlations between genotypic profiles/signatures and the response to standardized treatments of GBM using PDXs [164,165]. Another recent innovation is related to liquid biopsies in the context of cancer, in which cancer-relevant biomarkers are assessed in body fluids (e.g., blood, urine, cerebrospinal fluids, and ascites) [166,167]. Contrary to tumor tissue biopsy, liquid biopsies are noninvasive, allowing more frequent sample collection and monitoring of the tumor evolution longitudinally [2,168]. In particular for gliomas, the majority of studies have used blood samples and cerebrospinal fluid, and a residual number of reports evaluated urine samples [169]. Miller et al. (2019) [170] identified a genomic landscape of glioma based on cerebrospinal fluid that resembled the primary tumor, highlighting how liquid biopsies could be used to track glioma. Considering the intra- and inter-tumor heterogeneity of GBM, and its evolution, future studies should critically focus on how network analyses might assist the evaluation of omics data generated from liquid biopsies, which may facilitate and improve the level of examination of tumor dynamics, better predicting tumor progression and therapy responses. Molecular network models will also be pivotal in (near) single-cell analyses [171], depicting the multiple genetic patterns of individual cells that constitute a single tumor, potentially aiding in the selection and development of anti-GBM targeted therapies that may improve clinical responses, in a paradigm of precision medicine.

## 4. Conclusions

This narrative review summarizes the network-based strategies for network discovery and disease outcome prediction and the main findings when applied to GBM studies, along with the software developed and open challenges and future research opportunities. The role of network discovery has long been investigated in biological data, continuously challenged by the fast-growing technological advances. In the particular case of cancer genomics, advanced network-based algorithmic and computational tools are required to translate the vast amounts of high-dimensional and heterogeneous data into molecularly-informed clinical decisions. Trends in network discovery show a paradigm shift from association to causal discovery, with the premise that causal relationships disclose the direction of the association, therefore more meaningful for targeted interventions. While the development of causal inference methods rapidly evolves across several scientific domains, its application to GBM has been seldom investigated. Future GBM network-based studies might encompass a deeper focus of the causal nature of the biological mechanisms behind GBM, further contributing to our scientific understanding of this deadly disease and potentially improving clinical practice.

## Figures and Tables

**Table 1:** Glioblastoma databases and software tools used in the studies covered by the survey.

Resource	Website	Ref.
Genome projects		
The Cancer Genome Atlas (TCGA)	https://www.cancer.gov/tcga	[17]
Rembrandt	https://gdoc.georgetown.edu/gdoc/	[31]
Gene Expression Omnibus (GEO)	https://www.ncbi.nlm.nih.gov/geo/	[32]
NCBI Sequence Read Archive (SRA)	https://www.ncbi.nlm.nih.gov/sra	[33]
Glioblastoma Bio Discovery Portal	https://gbm-biodp.nci.nih.gov/	[34]
Cancer Cell Line Encyclopedia (CCLE)	https://portals.broadinstitute.org/ccle	[35]
Chinese Glioma Genome Atlas (CGGA)	http://cgga.org.cn/	[36]
Network Analysis		
DINGO	https://cran.r-project.org/web/packages/iDINGO/	[14]
WGCNA	https://horvath.genetics.ucla.edu/html/CoexpressionNetwork/	[37]
DiME	http://www.cs.bham.ac.uk/~szh/DiME	[38]
KINC	http://www.github.com/SystemsGenetics/KINC	[39]
WSNF	http://nugget.unisa.edu.au/Thuc/cancersubtypes/	[40]
CSPRV	https://github.com/yiangcs001/CSPRV	[41]
glmnet	https://cran.r-project.org/web/packages/glmnet/index.html	[42]
glmSparseNet	https://bioconductor.org/packages/release/bioc/html/glmSparseNet.html	[43]
ParallelPC	https://cran.r-project.org/web/packages/ParallelPC/	[44]
MSLCRN	https://github.com/zhangjunpeng411/MSLCRN	[45]
SYGNAL	http://www.synapse.org/#!Synapse:syn5907990	[46]
inTRINSiC	https://github.com/yunpengl9071/inTRINSiC	[47]
bnlearn	https://www.bnlearn.com/	[48]
Banjo	https://users.cs.duke.edu/~amink/software/banjo/	[49]

## Abbreviations

Banjo	Bayesian network inference with Java objects
BN	Bayesian network
CE	Community extraction
CLR	Context-likelihood-relatedness
CNA	Copy number aberration
CSPRV	Cancer subtype prediction using $R_{2}$
DAG	Directed acyclic graph
DEG	Differential expressed gene
DiME	Disease module extraction
DINGO	Differential network analysis in genomics
EMT	Epithelial-to-mesenchymal transition
EPoC	Endogenous perturbation analysis of cancer
GBM	Glioblastoma multiforme
GBM-BioDP	Glioblastoma Bio Discovery Portal
GEO	Gene Expression Omnibus
GMM	Gaussian mixture model
GO	Gene Ontology
GWAS	Genome-wide association study
HGG	High-grade glioma
IAMB	Incremental associated Markov blanket
IDA	Intervention calculus when the DAG is absent
iDINGO	Integrative differential network analysis in genomics
InTRINSiC	Integrative Modeling of Transcription Regulatory Interactions for
	Systematic Inference of Susceptibility in Cancer
KEGG	Kyoto Encyclopedia of Genes and Genomes
KINC	Knowledge Independent Network Construction
LGG	Low-grade glioma
TCGA	The Cancer Genome Atlas
CCLE	Cancer Cell Line Encyclopedia
CGGA	Chinese Glioma Genome Atlas
EN	Elastic net
lasso	Least absolute shrinkage selection operator
lncRNA	Long non-coding RNA
MI	Mutual information
MSLCRN	Module-specific lncRNA-mRNA causal regulatory networks
NGS	Next-generation sequencing
PDX	Patient-derived xenograft
RNA-seq	RNA sequencing
scRNA-seq	Single-cell RNA sequencing
SNF	Similarity Network Fusion
SNP	Single-nucleotide polymorphism
SYGNAL	Systems Genetics Network Analysis
TAM	Tumor-associated macrophage
TCI	Tumor-specific causal inference
TF	Transcriptional factor
TRN	Transcriptional regulatory network
twiner	Twin networks recovery
WGCNA	Weighted gene coexpression network analysis
WSNF	Weighted similarity network fusion
